# Endocan in Cancers: A Lesson from a Circulating Dermatan Sulfate Proteoglycan

**DOI:** 10.1155/2013/705027

**Published:** 2013-03-28

**Authors:** Maryse Delehedde, Lucie Devenyns, Claude-Alain Maurage, Romain R. Vivès

**Affiliations:** ^1^Lunginnov, Campus de l'Institut Pasteur de Lille, 59000 Lille, France; ^2^Centre de Biologie-Pathologie-Génétique, Centre Hospitalier Régional Universitaire de Lille, 59037 Lille Cedex, France; ^3^Institut de Biologie Structurale Jean-Pierre Ebel, Unité Mixte de Recherche (UMR) 5075, CNRS-CEA-Université Joseph Fourier, 38027 Grenoble, France

## Abstract

As most proteoglycans exert their biological activities in the pericellular region, circulating Endocan has appeared since its discovery as an atypical dermatan sulfate proteoglycan, with distinctive structural and functional properties. Endocan is naturally expressed by endothelial cells, highly regulated in presence of proinflammatory and proangiogenic molecules, binds to matrix proteins, growth factors, integrin, and cells, and may be then considered as an accurate marker of endothelial activation. Consequently, Endocan expression has been associated with a growing number of pathological conditions where endothelium gets challenged and notably in highly vascularized cancers. In this context, Endocan has indeed been rapidly emerging as a promising tissue- and blood-based marker of the vascular growth and neoangiogenesis during cancer progression. Furthermore, very recent studies have reported an expression of Endocan by the tumor cells themselves. This highlights Endocan as a multifaceted molecule with a great interest for researchers and clinicians to better understand tumor development, from the bench to the clinics. With promising perspectives of clinical applications, Endocan thus appears as an exciting model for on going and future developments of proteoglycan-based approaches in cancer diagnostics and/or therapy.

## 1. Introduction

During the past 20 years, proteoglycans (PGs) have emerged as critical modulators of most major cellular processes, including cell proliferation, adhesion, and migration, and have been involved in many pathological conditions, such as inflammation, cancer, or infection [1–5]. These complex glycoproteins, which consist of a core protein bearing polysaccharide chains of glycosaminoglycans (GAGs), are abundantly found at the surface of cells and in extracellular matrices (ECMs). From there, they benefit from an ideal positioning to interact, either through their protein core or their GAG chains, with soluble signaling effectors (growth factors, cytokines, chemokines…) [6, 7], structural components of the ECM (fibronectin, collagens…) and membrane-associated proteins (receptors, integrins…) [8–11], or intracellular molecules (PKC, Syntenin, CASK/Lin-2…) [12, 13]. However, PG activities are not restricted to the control of local contacts and molecular interactions at the cell surface vicinity. Shedding of cell-surface PGs (particularly HSPG Syndecans and Glypicans), enabling release of soluble GAG-associated ectodomains, is known to play a key role in many physiopathological conditions, such as wound healing, inflammation, tumor progression, and infection [14–16]. Paracrine activities can also be exerted by free, soluble PGs, such as Endocan, a small, soluble dermatan sulfate proteoglycan (DSPG) expressed by endothelial cells and naturally found in the blood circulation of healthy subjects [17–20]. 

The history of Endocan originates from the discovery in 1996 of a soluble protein produced by endothelial cells: endothelial cell-specific molecule-1 (ESM-1) [21]. Posttranslational modification studies later led to the identification of a DS chain on ESM-1 protein core, which was consequently included to the PG family, under the new name of Endocan [17]. Since then, Endocan has rapidly emerged as a very distinctive PG, in many aspects: (i) Endocan is the one of very few circulating PG *sensu stricto*, (ii) in contrast with most ECM PGs, which are usually large, modular proteins that harbor several GAG chains, Endocan comprises a relatively small polypeptide associated to one single polysaccharide chain, and (iii) characterization of Endocan DS chain revealed uncommon structural features and saccharide composition [22]. Interestingly, increasing experimental evidence has reported that Endocan is overexpressed in cancer, sepsis, obesity, or inflammatory conditions [20, 21, 23–28]. Furthermore, blood levels of freely circulating PG have been associated with poor prognosis in several types of cancers (reviewed in [28]). The aim of this paper is to review the present knowledge about Endocan expression, structure, and activity, with a special focus on the recent developments that have been highlighting that Endocan, as an endothelial proteoglycan not only is a biomarker of neoangiogenesis but also appears today as a signature of the tumor progression when it is expressed by the tumor cells themselves.

## 2. Expression of Endocan: A Marker of Endothelial Activation?

Endocan is the product of one single gene, *esm*, located in the proximal region of chromosome 5 long arm (5q11.2) [21]. The *esm* gene is organized in 3 exons separated by 2 introns, encoding an 552 bp open reading frame. Transcriptional control is ensured by a 3888 bp promoter at the 5′ flanking region of *esm*, which features a typical TATA box and a number of putative transcriptional binding sites, including Ets, Hhex, and CRE-like motifs [29, 30]. Noteworthy, Ets and Hhex motifs have been involved in the regulation of genes encoding several angiogenic and ECM remodeling factors, such as VEGF receptors-1 and -2, Tie-1 and -2, Neuropilin-1, or VE-cadherin [24, 29, 30]. Endocan mRNAs were first identified from cultures of human umbilical vein endothelial cells (HUVECs) [21], but expression of Endocan was subsequently reported in a large panel of cultured human endothelial cells, such as endothelial cells from the dermal microvasculature, the coronary and pulmonary arteries, or capillaries from adipose tissues [29, 31]. Surprisingly, Endocan could not be detected in highly vascularized organs, such as brain, heart, pancreas, or liver [21, 31], suggesting that Endocan expression may be characteristic of activated rather than resting endothelial cells. In support to this, a recent study reported Endocan expression during the endothelial-mesenchymal transition process, suggesting a role during arterial wall remodeling [32]. *In vivo*, Endocan is detected with an average concentration of ~1 ng/mL in the serum healthy patients [18] However, as detailed below, levels significantly increase in patients with septic shock [18] or cancers such as lung [18], kidney [33], or colon [34] cancers. 

Expression of Endocan is regulated by a number of cytokines and growth factors. Tumor necrosis factor-*α* (TNF-*α*), interleukin-1 (IL-1), transforming growth factor-*β*1 (TGF-*β*1), fibroblast growth factor-2 (FGF-2), and vascular endothelial growth factor (VEGF) have been shown to induce Endocan expression *in vitro* [35–40], while interferon-*γ* (IFN-*γ*) inhibits TNF-*α* induced upregulation of Endocan [21]. Signaling pathways involved in the control of Endocan expression remain poorly defined. One of the first studies on this topic came from Daly and collaborators, who reported that Endocan (as angiopoetin 2) is a downstream target of the endogenous transcription factor FKHR that regulates many genes associated to vascular destabilization/remodeling [41]. Using pharmacological compounds, Abid and collaborators investigated intracellular pathways involved in VEGF-induced secretion of Endocan in cultured endothelial cells [42]. VEGF induction of Endocan mRNA was blocked by BIM (PKC inhibitor) but not PD98059 (MEK1/2 inhibitor). In contrast, preincubation of HUVEC with LY294002 (PI3 K inhibitor) or with PMA (PKC activator) resulted in increased basal levels of Endocan mRNA. VEGF-mediated induction of Endocan was therefore found to be positively and negatively regulated by PKC/NF*κ*B and PI3 K/AKT/FKHRL1 signaling pathways, respectively. This was further supported by Rennel and collaborators, who also reported that VEGF-A induction of Endocan expression in cultured endothelial cells was regulated via the PI3 K pathway. Furthermore, they showed that PI3 K inhibitor LY294002 alone caused an increase in Endocan transcripts to levels higher than those induced by VEGF [39]. Noteworthy, recent studies have suggested that Endocan expression may regulate cell survival in cancer cells via the NFKB pathway [43, 44]. However, a truncated form of Endocan has also been identified in HUVEC [31]. This variant, resulting from alternative splicing of exon 2, is not secreted and not glycosylated (deletion of the GAG attachment site) [18, 31]. 

## 3. Endocan: An Atypical Structure for an Atypical Proteoglycan

**Figure 1:**
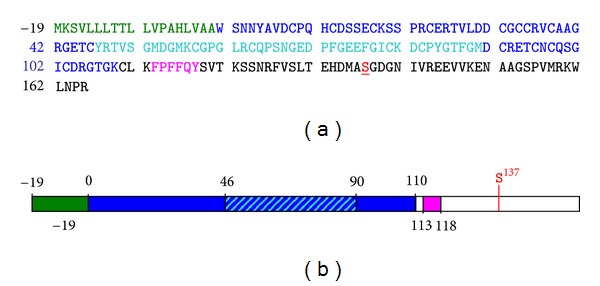
Structure of Endocan protein core. Amino acid sequence (a) and domain organization (b) of Endocan protein core are shown according to the following color code: signal peptide (in green), cysteine-rich region (dark blue) including the EGF-like domain (light blue), phenylalanine-rich region (pink), and C-terminal region (white) including DS attachment residue S^137^ (red and bold/underlined).

Endocan is one of the rare circulating PGs that have been identified, along with bikunin [45] and macrophage colony stimulating factor-1 (MCSF-1) [46], which are both CSPGs. Structurally, Endocan is composed of a 165 amino acid core protein bearing one single DS chain (Figure 1). The protein moiety features an N-terminal cysteine-rich region (W^1^-K^111^) that includes an EGF-like domain (Y^46^-M^90^), a phenylalanine-rich region (F^113^-Y^118^), and a C-terminal region (S^119^-R^165^) that comprises the S^137^G^138^ dipeptide, where attachment of the DS chain occurs. Surprisingly, Endocan shares no obvious homologies with other extracellular members of the PG family. Endocan does not include leucin-rich repeats, which are the signature of small leucine-rich protein (SLRP) PGs, nor C-type lectin-like domains that are typically found in hyalectans [11]. EGF-like domains are also present in hyalectan PGs as well as in HSPGs, such as Perlecan or Agrin [11]. In Perlecan, such domains have been suggested to play a role in protein oligomerisation. However, no precise function has been associated yet with the single EGF-like domain found in Endocan. 

In contrast, 15–28% of sequence identity can be found between the cystein-rich N-terminal region of Endocan and members of the insulin-like growth factor binding protein (IGFBP) family. However, unlike these proteins, Endocan does not bind to IGF [21, 47]. In addition, Endocan sequence shows some homology with human *β*2 integrin, fibrillin-1, laminin-*β*2, and procollagen *α*2 [21]. Finally, a 74% sequence homology was found with PG25, a protein isolated from rat pineal gland [48]. PG25 has therefore been categorized as Rat Endocan, thereby representing the first Endocan ortholog to be identified at the protein level. Expression of Endocan orthologs has also been identified by southern blot in mouse, rabbit, dog, cow, and monkey, with a high degree of gene conservation [21, 29, 48]. 

**Figure 2:**
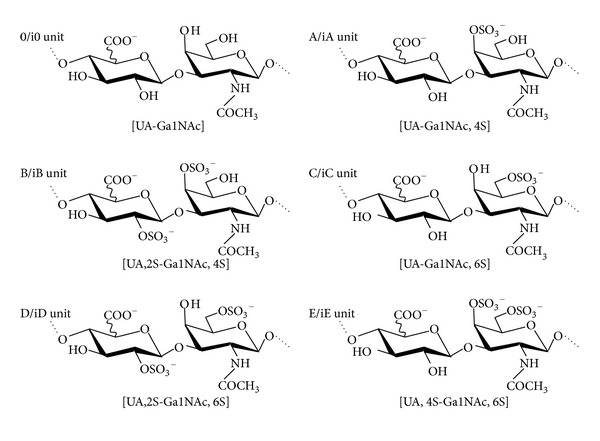
Structure of typical disaccharide units found in CS/DS. CS/DS disaccharide units are composed of an N-acetylated glucosamine (GalNAc) associated to an uronic acid (UA) through a *β*1-3 linkage, with variations in sulfation pattern as indicated. UA is a glucuronic acid for 0-E units (CS disaccharides) and an iduronic acid for i0-iE units (DS disaccharides).

The saccharide moiety of human Endocan is represented by as single chain of dermatan sulfate (DS) [17]. DS is a member of the GAG family, which include glucosaminoglycan HA, heparin and HS, and galactosaminoglycans CS and DS [49, 50]. GAGs are complex, linear polysaccharides, constituted of a repeating disaccharide unit comprising an *N-*substituted hexosamine and an uronic acid. In galactosaminoglycans, the hexosamine is an *N-*acetylgalactosamine, which is associated with either glucuronic acid (GlcA) for CS or GlcA and a variable proportion of its C5-epimer iduronic acid (IdoA) for DS. In addition, this saccharide backbone is modified further by addition of *O*-sulfate groups: at C-2 of the uronic acid and at C-4/C6 of the galactosamine. Resulting sulfation patterns have led to a classification of CS disaccharide units as 0 units standing for GlcA-GalNAc, A units for GlcA-GalNAc,4S, B units for GlcA,2S-GalNAc,4S, C units for GlcA-GalNAc,6S, D-units for GlcA,2S-GalNAc,6S, and E-units for GlcA-GalNAc,4,6S (Figure 2). For DS comprising a IdoA instead of GlcA, i0, iA, iB, iC iD, and iE units have been defined accordingly. Both saccharide backbone and sulfation pattern provide the structural basis of CS/DS large interactive properties. IdoA residue confers functional specificities to DS [51–53], most probably by enhancing flexibility of saccharide motifs [54]. Furthermore, increasing evidence has shown that specific CS/DS sulfation patterns were involved in precise biological functions and that variations of these sulfation patterns can be associated with physiological changes and pathological developments [55–57]. 

In contrast with most extracellular PG, one single GAG chain is found attached to Endocan protein core. Early studies reported partial susceptibility to chondroitinase B, thereby identifying Endocan chain as of the DS type [17]. Since then, its structural features and binding properties have been extensively studied [17, 22]. The DS chain Endocan expressed by endothelial cells is ~15 kDa in size (e.g., ~32 disaccharides), which is relatively shorter than CS/DS chains found on PGs. This unique GAG chain includes a significant proportion of IdoA residue (31%) that are distributed along the polysaccharide chain, either as alternating GlcA/IdoA-containing disaccharide sequences or in short clusters of 2-3 disaccharides [22]. Most interestingly, when compared to CS/DS from various sources [55, 57], disaccharide analysis of Endocan DS revealed a higher proportion of nonsulfated disaccharides (10–12% of ΔHexA-GalNAc) and of the disulfated B, D, and E units that are rather rare in mammals. As highly sulfated domains are usually involved in GAG/protein interactions, it is speculated that the presence of such disaccharide may be critical for Endocan biological properties.

## 4. Binding Properties of Endocan

As many PGs, biological properties of Endocan depend on ligand interactions involving either its protein core or its DS chain. *In vitro*, Endocan protein core binds to lymphocytes and monocytes through a high affinity interaction with the integrin CD11a/CD18, also called lymphocyte function-associated antigen-1 (LFA-1) [19]. Endocan inhibits LFA-1/ICAM-1 interaction, thereby affecting leukocyte adhesion and migration in tissues [19, 58]. Endocan DS chain has been shown to bind and activate hepatocyte growth factor (HGF) *in vitro* [17, 22]. HGF is a multifunctional growth factor that can bind to both HS and DS GAGs and promotes, through interaction with its specific receptor Met, motility and proliferation of many different cell types cells [59, 60]. Importantly, Endocan variant lacking the DS chain failed to induce HGF activation, thus confirming the role played by the GAG chain in this process [17]. Furthermore, biosensor analysis reported a *K*_*d*_ of ~3–6 nM for HGF/Endocan interaction [17], which is well in the 0.2–20 nM range of previously published affinity values for HGF binding to free HS and DS chains [51, 61, 62]. High affinity interaction of GAGs with HGF is critical for its activation of the Met receptor, which plays major roles in many physiopathological processes, including development, wound healing, and tumor progression [63–65]. Through its ability to promote HGF activity, Endocan may therefore be involved in some of these processes *in vivo*. 

In addition, Endocan has been shown to promote the mitogenic and migratory activities of VEGF-A and -C *in vitro* [66]. Finally, DS is known to bind to a multitude of signaling and adhesion molecules, including L- and P-Selectins, matrix proteins such as fibronectin, chemokines/cytokines RANTES, SDF-1*β*, Il-8, MCP-1, IFN-*γ*, and PF-4, and growth factors Midkine, Pleiotrophin FGF-2, and -7 [24, 57], leaving open the question of a possible interaction of these ligands with Endocan.

## 5. Vascular Endocan as a Biomarker of Neoangiogenesis in Cancer

Angiogenesis is a key event in many cancer types. This process gives rise to a new blood supply so that an emerging tumor shifts from a localized stage to an aggressive behavior [67, 68]. Pertinent biomarkers of angiogenesis are urgently needed to detect this shift in order to apply the best personalized treatment to the patient. The major growth factors and cytokines involved in vascular growth have been failing as biomarkers of the progression in tumor development. Endocan, as a PG secreted by endothelial cells when activated, has appeared over the years as a promising tissue-based and also blood-based marker and we will detail later the most recent literature evidences.

The release of Endocan by cultured endothelial cells has been largely described to be strongly upregulated in presence of proangiogenic molecules such as VEGF, FGF-2, and VEGF-C that are critical mediators involved in angiogenesis (i.e., formation of blood vessel), lymphangiogenesis (i.e., formation of lymphatic vessel), and cancer progression (spreading and metastasis) [23, 28, 35, 36, 39, 66]. In addition, Endocan has been shown to directly promote the mitogenic and migratory activities of VEGF-A and -C on cultured endothelial cells [66]. Interestingly, siRNA silencing of Endocan expression inhibits VEGF-A/-C induced proliferation of lymphatic endothelial cells [66]. Furthermore, addition of recombinant Endocan protein to Endocan siRNA-transfected endothelial cells restores the stimulatory effect of VEGF-A on migration [66], hence suggesting a role of Endocan in lymphangiogenesis. Endocan expression is also induced in cultured endothelial cells undergoing tube formation as an assay to assess angiogenesis and vasculogenesis *in vitro* [31]. In animal models, Endocan gene is one of the main genes involved in the angiogenic switch occurring during retinal neovascularization induced by hypoxia [69]. A link has been recently established between Endocan and the “tip cells,” the specialized subset of endothelial cells known to mediate vessel growth in neoangiogenesis [70, 71]. These tip cells are the sensor cells at the end (the tip) of the growing new vessels, which lead the building of the tumor vascular network required for tumor progression. The “stalk” cells (constituting the vessel to be) are not or less expressing Endocan [71], which may suggest that Endocan will decrease with vascular regression. Taken together, all these experimental data highlight Endocan as a marker of endothelial cell activation during the growth of new vessels required for tumor progression. 

**Figure 3:**
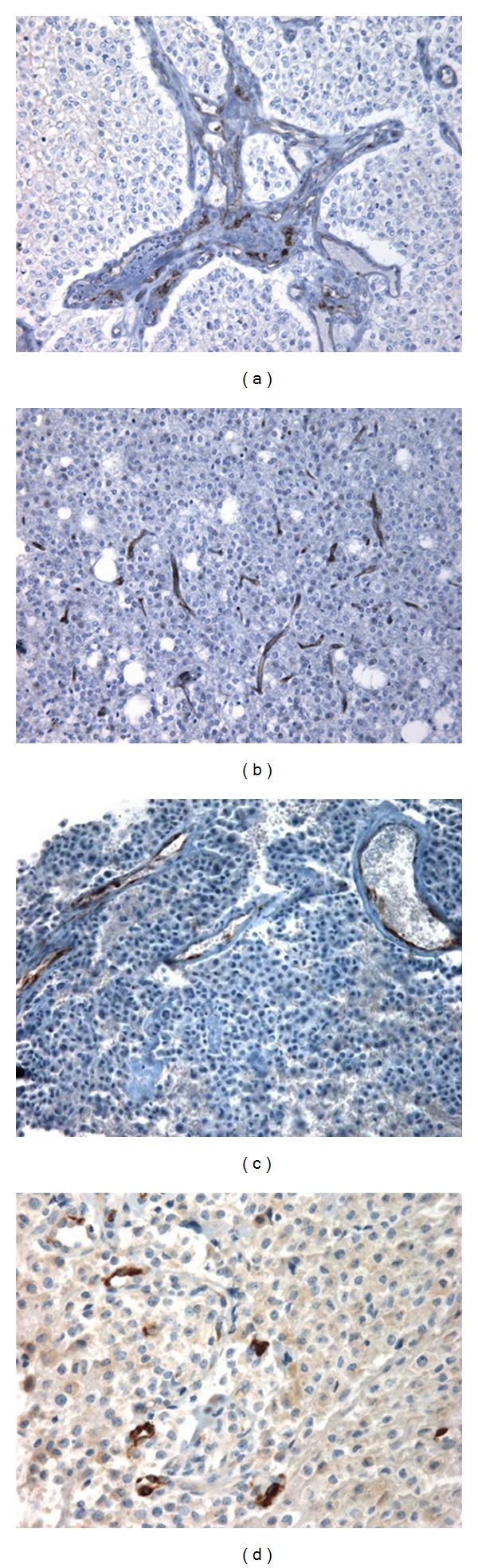
Endocan is expressed by endothelial cells during neoangiogenesis. (a) Endocan-immunopositive endothelial cells are always observed in multilayered endothelium characteristic of tumor vessels of the high-grade gliomas also called glioblastoma. (b) In brain tumor, Endocan is detected by immunohistochemistry even in one isolated but activated endothelial cells as an early event of neoangiogenesis. (c) Endocan is expressed by activated endothelial cell from small and large vessels in ACTH adenoma (adrenocorticotropic hormone adenoma from the pituitary). (d) Endocan reactivity is also observed even in small vessels and sparse endothelial cells in GH adenoma of the pituitary. Courtesy from C. A. Maurage.

Data obtained from cancer tissue samples strengthen all that experimental evidence of Endocan as a relevant biomarker for clinics. By immunohistochemistry, Endocan is visualized in the cytoplasm of endothelial cells of the newly growing tumor vessels (Figure 3). In the literature, a marked cytoplasmic expression of vascular Endocan has been then described inside the tumor vessels in different types of highly vascularized tumors such as cancer of lung [20, 23], brain [35, 36], colon [35, 44, 72], liver [73, 74], kidney [33], the pituitary [75], stomach [35, 76], prostate, and testis [35].

Endocan detection may be today the most pertinent tool to discriminate the “good” (the resting vessel in normal tissue), the “bad” (the emerging vessel dedicated tumor angiogenesis), and the “ugly” (the proliferating multilayered capillary as seen in highly aggressive tumor of the brain). Indeed, Endocan is detected in the vessels primarily activated by VEGF [23, 74]. Glioblastoma, which is the most aggressive tumor of the brain in adults, is a VEGF-driven tumor characterized by extensive angiogenesis and proliferating multilayered capillaries. In glioblastoma, Endocan expression is always associated with the abnormal vasculature reflecting neoangiogenesis [35, 36], as shown in Figure 3(a). In contrast, Endocan is never detected in low-grade gliomas (grade I and grade II) and in the cerebral cortex distant from the tumors [35, 36]. Another cancer of particular interest is the clear cell renal carcinoma (CCRC) that usually shows a huge neoangiogenesis driven by the VHL (von Hippel Lindau) mutation, and that is currently mainly treated by antiangiogenic drugs. Endocan is reported to be clearly expressed in all the vessels of the CCRC, inside the tumor sample, but is never detected in the vessels of other types of kidney tumors [33]. Furthermore, in the same tumor section, Endocan as a biomarker of activated endothelial cells helps to distinguish the resting from the emerging capillaries. Microscopically, the quantification of the density of capillaries per mm² (also called microvascular density: MVD) in a cancer sample is not by itself relevant to show the angiogenic process if all vessels (normal and pathologicals) are taken into account. Interestingly, the Endocan immunolabeling (Endocan-MVD) was shown to correlate more strongly with aggressiveness (and shortened survival) for the first time in liver carcinoma than an MVD measured using a pan-endothelial surface marker such as CD34, as described for the first time in liver carcinoma [74]. Since then, other studies in colon, liver, and pituitary tumors have described that MVD stained by Endocan is correlated with microscopic venous invasion, VEGF expression, recurrence, and/or bad prognosis [43, 73, 75, 77].

The immunodetection of Endocan in tissues may help to reveal an aggressive behavior and/or recurrence according to the type of tumors. For instance, Endocan immunoreactivity within endothelial cells clearly correlates with a shorter survival in glioblastoma patients [36]. Similarly, Endocan expression by endothelial cells in hepatocarcinoma is associated with unfavorable prognostic features [73]. In pituitary tumors, a strong association between Endocan immunoreactivity of the vessels and recurrence has been recently described in 107 patients with a long follow-up [75]. Endocan immunodetection in activated colorectal cancer endothelial cells has also been reported as an independent prognostic factor for disease recurrence and worse survival outcome [44].

Since Endocan expression reflects endothelial activation occurring during neoangiogenesis of tumors, dosage of the secreted protein in the blood circulation could also provide a valuable read out of tumor progression and of patient response to drugs. Secreted Endocan is naturally found at low levels in the sera from healthy subjects [17]. Levels of circulating Endocan are found to be from 3- to 10-fold higher in the sera of patients with renal carcinoma compared to healthy subjects [33]. Accordingly, increased blood levels of Endocan are detected in patients with lung cancers, with the highest levels of Endocan in patients with poor prognosis [20, 23]. Endocan is also a potential serum marker for early detection of colorectal cancer [34] and also of liver carcinoma [43]. These different studies have all highlighted the potential of Endocan as a blood-based biomarker to assess endothelial activation in angiogenesis-driven tumorigenesis. Indeed, Endocan as a biomarker could also be of great interest for the selection of patients susceptible to angiogenesis-directed therapies or for identifying the probability of resistance, the major cause of failure of cancer treatment. Interestingly, treatment of cultured endothelial cells by antiangiogenic drugs (such as anti-VEGF antibodies or small drugs) abolishes the VEGF-induced secretion of Endocan [23, 33, 42]. For instance, Endocan expression was shown to decrease under treatment with specific VEGF receptor-2 kinase inhibitors [78] or the addition of Sutent (a multityrosine kinase inhibitor) directly into the culture medium completely abolished the secretion of Endocan induced by VEGF from HUVEC cells [33]. Furthermore, a combined blockade of VEGFR-2 and VEGFR-3 by specific antibodies completely prevented VEGF-C-mediated induction of Endocan expression in endothelial cells [66].

Other PGs have been proposed as potential biomarkers of tumor progression, such as the proteolytically shed ectodomains of Syndecan-1 and Glypican-3. For instance, the level of shed Syndecan-1 is increased in sera of patients with lung cancer, myeloma, or liver cancer and was associated with bad prognosis [79–81]. However, their use as diagnostic tools remains controversial. As such, further investigations will be required to fully assess the pertinence of Endocan as biomarker. No other molecule is currently available to assess endothelial activation and/or dysfunction during tumor progression and Endocan could represent a pertinent blood based marker that helps detect neoangiogenesis and response of treatment. 

## 6. Endocan Is Also Expressed by Tumor Cells: A Signature of Tumor Progression?

**Figure 4:**
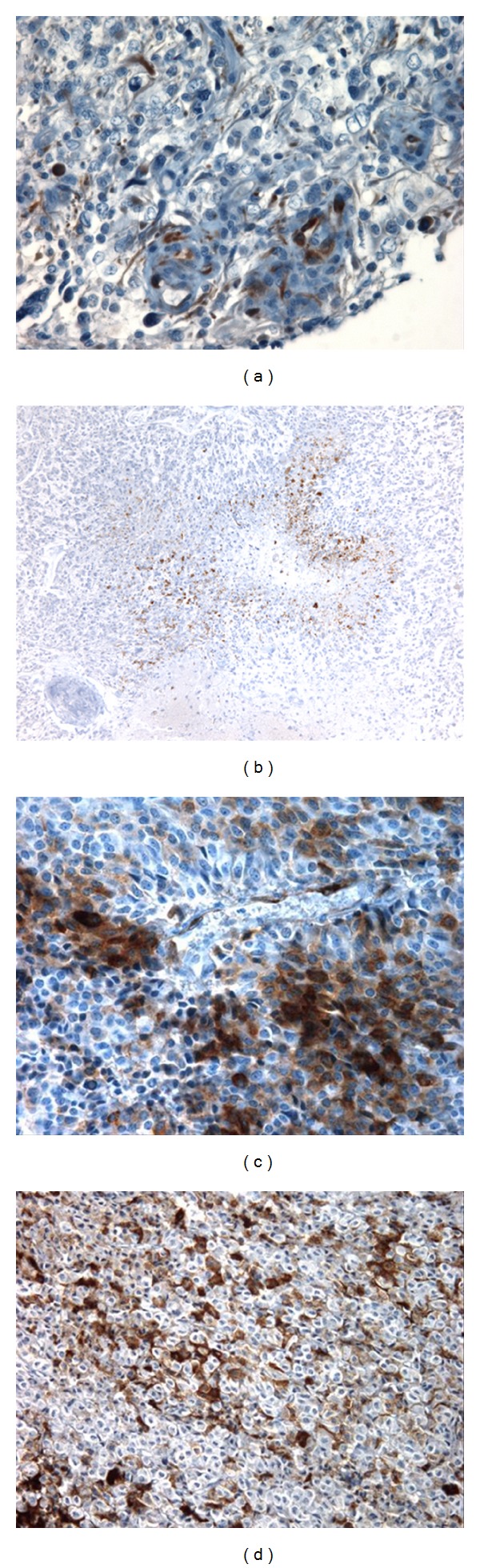
Endocan is expressed by tumor cells in cancer progression. (a) In the same tumor section, Endocan positive cells are detected in tumor cells and in endothelial cells from multilayered vessel of glioblastoma. (b) In glioblastoma, Endocan is always expressed in the palisading cells that are tumor cells in area around necrosis. (c) Immunostaining of tumor cells in pituitary adenoma (ACTH producing tumors). (d) Immunostaining of tumor cells in pituitary adenoma (GH producing tumors). Courtesy from C. A. Maurage.

Study of Endocan in cancer samples took another twist, when expression of the PG by the tumor cells themselves has been reported by immunohistochemistry [36]. Surprisingly, Endocan is always present in the cytoplasm of the tumor cells of high grade gliomas also called glioblastoma (Figure 4(a)). Furthermore, Endocan tumor immunolabeling was always associated with the typical hypoxic palisades of poorly differentiated tumor cells in glioblastoma as shown in Figure 4(b) [36]. Another recent example of Endocan expression in tumor cells came from the pituitary where Endocan was detected in both endothelial and tumor cells [75] as shown in Figures 4(c) and 4(d).

The question was then raised about the role of Endocan expression in tumor cells themselves. Going through the literature, an early study had already presented Endocan as a protumorigenic molecule when expressed in tumor epithelial cells [20]. When colon tumor HT29 cells (that are naïve for Endocan) were manipulated to express Endocan, they became more tumorigenic in a mouse xenotransplantation model [20]. Interestingly, when overexpressed in tumor cells, only the clones expressing the DSPG (i.e., fully glycanated Endocan) enhance tumorigenesis, thereby underlining the role of the DS chain in the protumorigenic activity of Endocan [20, 82]. In addition, increased Endocan expression in tumor cells was associated with enhanced invasiveness and metastasis in prostate cancer cells [83]. Endocan was upregulated by 26.5-fold in rat prostate MAT-LyLu cell lines with highly metastatic phenotype compared to nonmetastatic cells derived from Dunning R3327 cells [83]. 

Further investigations will be needed to clarify the role(s) of Endocan expression in the tumor cells, but the first hints arose when Endocan was described in cultured tumor cell lines as one of the genes associated with two of the major events in tumor progression: the angiogenic switch and the vascular mimicry of tumor cells. First, the angiogenic switch has been described as the key step that determines the development from a dormant and localized tumor into an aggressive tumor able to disseminate and spread over the body [84]. The *esm* gene encoding Endocan appears as a signature of the angiogenic switch from dormant to aggressive phenotype of breast, brain, osteosarcoma, and liposarcoma tumor cell lines [84, 85]. Interestingly, levels of Endocan expression correlated with tumor aggressiveness, with elevated levels of Endocan reported in the aggressive clones generating angiogenic fast-growing tumors in xenografted animal models, and low levels of Endocan in clones leading to dormant tumors [84, 85]. Increase of Endocan gene expression was notably high (30-fold increase) in glioblastoma and liposarcoma models [84]. Interestingly, the tumor cell lines release *in vitro* Endocan as a DSPG [36, 84] and the secretion of Endocan by these tumor cells was shown to be upon regulation of proinflammatory molecules such as TNF-*α* and of proangiogenic growth factors such as FGF-2 as previously described for endothelial cultured cells [24].

The expression of Endocan in tumor cells could also be explained by the mechanism described as the vasculogenic mimicry. Vasculogenic mimicry is defined as the unique capability of highly aggressive tumor cells—but not of poorly aggressive cells—to express endothelium-associated genes, to form patterned, tubular networks in three-dimensional culture, which could convey blood plasma and red blood cells and therefore mimics the vasculogenic networks formed by endothelial cells [86]. When involved in vasculogenic mimicry, skin melanoma tumor cells have been shown to strongly express endothelial cell-associated genes including Endocan [87, 88]. Indeed, Hendrix and collaborators were the first to describe Endocan to be one of the most upregulated genes *in vitro* (44-fold increase) in highly aggressive skin and uveal melanoma cells compared to poorly aggressive melanoma cells [87, 88]. The vasculogenic mimicry of tumor cells may then facilitate tumor perfusion and consequently the tumor metastasis. This process has been already observed in various types of cancers from prostate, bladder, breast, ovary, and brain [89, 90]. The tumor cell plasticity seems to trigger vascular mimicry and the aggressive tumor cells may revert to an undifferentiated stem cell-like phenotype, with a pluripotent gene expression pattern. In glioblastoma, the ability of these cancer stem-like cells to directly contribute to the tumor vasculature by endothelial cell differentiation has largely been described [89, 91–93]. Endocan is always expressed in tumor cells in glioblastoma, particularly in the palisade area and was never observed in low grade tumors [36, 84, 85]. The potential of Endocan as a marker of vascular mimicry, of tumor plasticity, or its potential role in cancer stem-like cells for tumor progression still needs to be further investigated.

## 7. Conclusion

**Figure 5:**
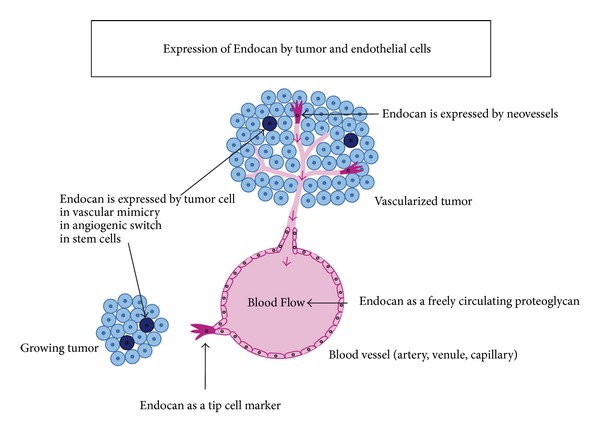
Expression of Endocan by endothelial and tumor cells.

Endocan is therefore a unique circulating PG that appears today as a molecule of versatile interest in the study of tumor progression (Figure 5). First, increasing evidence underlines Endocan as a pertinent biomarker of neoangiogenesis in cancers. The dermatan sulfate chain seems critical to bear the roles of Endocan in vascular and tumor growth. There is now an urgent need to assess, in a larger trial, the clinical value of Endocan as a tissue- and blood-based biomarker in cancers where neoangiogenesis is the main driver of tumor progression. As no suitable biomarker has been yet validated in clinics to identify the patients clearly susceptible to benefit of angiogenesis-directed therapies, clinical studies should be set up to evaluate Endocan as a predictive biomarker for antiangiogenic therapies. Furthermore, the recent identification of Endocan expression by tumor cells represents a novel and major development in the study of this PG. Indeed, the large gene expression studies from microarrays that originally revealed the potential of Endocan as a gene of bad prognosis signature in various types of cancers did not take into account the part of the endothelial versus the tumor expression of Endocan. This particular expression of Endocan in tumor cells will need further investigation to better understand the prognostic value of Endocan expression in tumor versus endothelial compartment. 

## Abbreviations

ESM-1: Endothelial cell-specific molecule-1
ECM: Extracellular matrix
PG: Proteoglycan
GAG: Glycosaminoglycan
CS: Chondroitin sulfate
DS: Dermatan sulfate
HS: Heparan sulfate
KS: Keratan sulfate
HA: Hyaluronic acid
TNF-*α*: Tumor necrosis factor alpha
HGF/SF: Hepatocyte growth factor/scatter factor
VEGF: Vascular endothelial growth factor
FGF-2: Fibroblast growth factor 2
HUVEC: Human umbilical vein endothelial cells
GalNAc: N-Acetyl galactosamine
GlcA: Glucuronic acid
IdoA: Iduronic acid.
